# Correlation between genetic alterations and growth of human malignant glioma xenografted in *nude* mice

**DOI:** 10.1038/sj.bjc.6601466

**Published:** 2003-12-09

**Authors:** P Leuraud, L Taillandier, L Aguirre-Cruz, J Medioni, E Crinière, Y Marie, A M Dutrillaux, M Kujas, A Duprez, J-Y Delattre, M-F Poupon, M Sanson

**Affiliations:** 1 INSERM U495, Laboratoire de Biologie des Interactions Neurones-Glie, Groupe hospitalier Pitié-Salpêtrière AP-HP, Paris, France; 2 Laboratoire d'anatomopathologie et de microchirurgie expérimentale, Faculté de Médecine, Vandoeuvre-les-Nancy, France; 3 Unité Fonctionnelle de Biostatistiques, Groupe hospitalier Pitié-Salpêtrière AP-HP, Paris, France; 4 Centre d'Investigations Cliniques, Hôpital Saint Louis AP-HP, Paris, France; 5 FRE 2485 CNRS, Institut Curie, Paris, France; 6 Laboratoire de neuropathologie R Escourolle, Groupe hospitalier Pitié-Salpêtrière AP-HP, Paris, France; 7 Fédération de Neurologie Mazarin, Groupe hospitalier Pitié-Salpêtrière AP-HP, Paris, France; 8 Unidad de Investigación del Sistema Nervioso, Instituto Nacional de, Neurología y Neurocirugía de México, México D. F.

**Keywords:** oligodendroglioma, xenograft, EGFR, preclinical models

## Abstract

In order to develop preclinical models of malignant astrocytomas and oligodendrogliomas, a series of 54 resected gliomas (37 from oligodendroglial lineage and 17 from astrocytic lineage) were xenografted subcutaneously into *nude* mice. Molecular alterations commonly observed in gliomas subtypes, including LOH 1p and 1q, LOH 19q, LOH 10p and 10q, LOH 9p, TP53 and PTEN mutations, EGFR amplification, CDKN2A homozygous deletion and telomerase reactivation were systematically screened in the original and xenografted tumours. In all, 23 gliomas grew in *nude* mice. The most anaplastic tumours were selected as shown by pathological and molecular studies of the original tumour as well as shorter survival in patients whose tumours were successfully grafted. Comparison between the two growth profiles showed that 10q LOH and EGFR amplification gave a tumorigenic advantage. With a few exceptions, the genetic pattern was remarkably stable before and after growth in *nude* mice. These results suggest that subcutaneous xenografts are useful and reproducible models to analyse the molecular profile of malignant astrocytoma and oligodendroglioma. This represents the first step to improve our understanding of the correlations between molecular alterations and response to standard or experimental therapies.

Over the last decade, important advances have been made in the understanding of the molecular pathways involved in the progression of astrocytomas and oligodendrogliomas (Louis *et al*, 2001). Recent data also suggest that acquired genetic alterations, such as LOH 1p–19q, could be important predictors of prognosis and response to therapy (Cairncross *et al*, 1998; Smith *et al*, 2000; Ino *et al*, 2001). Unfortunately, the lack of clinically relevant models of gliomas seriously affects fine analysis of the correlation between genotype and treatment response. This is particularly clear for oligodendrogliomas, which are very difficult to maintain in *in vitro* cultures (Merrill and Matsushima, 1988; Post and Dawson, 1992; McLaurin *et al*, 1995). In this setting, well-characterised xenografts of gliomas could be of help, as specified by the recommendations of the European Medicines Evaluation Agency (EAEMP, 1999) and the EORTC Laboratory Research Division (EORTC LRD, 2001). However, it remains to be demonstrated that astrocytic and oligodendroglial tumours can grow reproducibly in *nude* mice while maintaining the same profile of genetic alterations as in the original tumour.

## MATERIAL AND METHODS

### Tumour classification

A set of 54 gliomas was surgically resected at the Pitié-Salpêtrière hospital, Paris and the Centre Hospitalier Régional, Nancy. An informed consent was obtained for all patients. After pathological review according to the current WHO guidelines (MK) (Kleihues and Cavenee, 2000), except for the addition of a recently individualised subgroup of glioblastoma with an oligodendroglial component (GBMO) (He *et al*, 2001), the tumours were classified as oligodendroglioma (one O), mixed gliomas (two OA), anaplastic oligodendroglioma (29 AO), anaplastic oligoastrocytoma (one AOA), four GBMO and finally a subset of 17 glioblastoma (GBM) that was used as a control group.

### Xenograft protocol

Each tumour was xenografted subcutaneously into the scapular area. Following a maximal delay of 2 h after surgical resection, small fragments (∼50 mm^3^) of tumour tissue were xenografted onto at least three anaesthetised *nude* mice (Swiss *nu/nu*) aged 6–8 weeks. Before xenograft, tumours were kept in foetal calf serum-free DMEM (Sigma-Aldrich, St Louis, MO, USA). The mice were maintained under clean room conditions and received sterile rodent food and water *ad libitum*. Their care and housing were in accordance with institutional guidelines as put forth by the Ministère de l'Agriculture et de la Forêt, Direction de la Santé et de la Protection Animale, Paris, France and the standards required by the UKCCCR guidelines (Workman *et al*, 1998).

The mice were kept alive for a maximum of 12 months until the growth of the xenografts. The tumours were measured once a week in two perpendicular dimensions and their volumes were estimated using the formula (width)^2^ × (length)/2. Latency was defined as the time between first transplantation and the appearance of a palpable tumour. The doubling time was calculated once it became stable, that is, usually after the fifth passage. The mice were killed when the tumour volume reached 2000 mm^3^ and the xenografts were maintained by serial transplantation.

## DNA EXTRACTION

Paired blood and tumours could be obtained for 31 initial tumours, blood could not be obtained for 12 tumours, and unfortunately tumour samples were not fresh frozen in 11 cases. Blood DNA was extracted using the Nucleon BACC3 DNA Extraction kit (Amersham Bioscience, Piscataway, NJ, USA) and tumoral DNA was extracted using the QIAamp DNA minikit, as described by the manufacturer (Qiagen, Venlo, NL, USA).

### Microsatellite analysis for loss of heterozygosity (LOH) on chromosomes 1, 9p, 10 and 19q

Blood and tumour DNA were screened for LOH on chromosome 1p, using the following polymorphic markers: D1S450, D1S2667, D1S234, D1S255, D1S2797, D1S2890, on chromosome 1q using D1S2878, D1S249, D1S2785 markers. On chromosome 9p LOH was screened, using the following markers: D9S286, D9S168, D9S1870, D9S156, D9S1687, spanning the region located near CDKN2A. On chromosome 10p, LOH was screened using the following markers: D10S249, D10S189, D10S547 (near hTR repressor), D10S585 (near hTR repressor), D10S548, D10S204, on chromosome 10q using D10S537, D10S219, D10S1744 (near PTEN), D10S541, D10S579, D10S1755, D10S1671, D10S597, D10S1693, D10S209, D10S587 (near DMBT1), D10S1723, D10S212, D10S537, D10S541, D10S597, D10S1693, D10S212 markers spanning the region located between 10q21.22 and 10qter. And for chromosome 19q, LOH was screened using the following markers: D19S425, D19S219, D19S888, D19S412, D19S418. One of the primers was labelled with the Hex, Fam or Ned fluorochromes (Applied Biosystems, Foster City, CA, USA). The samples were run on an automatic sequencer and analysed with the Gene Scan program (Applied Biosystems, Foster City, CA, USA).

### Screening of the *PTEN/MMAC1* and TP53 gene mutations

*PTEN*/*MMAC1* mutations were screened by the denaturing gradient gel electrophoresis (DGGE) technique in the entire coding sequence of the nine exons and their corresponding splice junctions using previously described primers (Zhou *et al*, 1999). TP53 mutations were screened by the DGGE technique for exons 5–8 and their using previously described primers (Hamelin *et al*, 1994). DNA showing altered DGGE profiles were sequenced bidirectionally using the Perkin Kit and sequencer. When a DNA variant was found, the corresponding blood DNA was sequenced in order to differentiate somatic events from constitutional variants (polymorphism or germline mutation).

### Screening of EGFR gene amplification and P16/CDKN2A gene homozygous deletion

EGFR amplification was screened by semiquantitative PCR using primers and protocol described previously (Hunter *et al*, 1995). P16/CDKN2A homozygous deletions were screened by semiquantitative PCR using primers and protocol previously described (Walker *et al*, 1995).

### Screening of telomerase activity

Telomerase activity was screened by Telomeric Repeat Amplification Protocol (TRAP) technique using the TRAPeze kit (Intergen Co, Purchase, NY, USA) as described by the manufacturer.

### Statistical. analyses

Descriptive statistics for continuous variates are provided as mean, standard deviation, median, minimum and maximum. For categorical variates, frequency distribution, median, minimum and maximum are provided.

The relation between categorical variates is described using the χ^2^ method, or Fisher's exact test when the χ^2^ method is not appropriate.

Prognostic factors for tumour growth delay are identified using univariate analysis (log rank test) and multivariate analysis fitting Cox's proportional hazard regression models. All tests are considered significant at the 0.05 significance level. Odds ratios are presented with their 95% confidence interval.

Analyses were performed using the SAS/STAT software v8.0 (SAS Institute, Inc., Cary, NC, USA).

## RESULTS

### Patient characteristics and relationship with tumour growth

The 54 surgical samples were obtained from 33 men and 21 women, with a median age of 57. 4 years (range 20–77). The post-op median survival was 11.03 months after a median follow-up of 31.9 months (range 1–37.2).

Out of 54 tumours, 23 were established in *nude* mice (42.2%). In total, 14 were derived from oligodendroglial tumours (one AOA, nine AO, four GBMO) and nine from GBM. When a tumour was tumorigenic, it grew on at least 60% of mice at the first transplantation and on 80–100% mice on later transplantations.

None of the three low-grade gliomas were established in nude mice, while 23 out of 51 high-grade gliomas did (45%).

Out of 21 grade IV gliomas, 13 were established in *nude mice* (62%), while 10 out of 30 grade III did (33%) (*P*=0.0047).

Patients, whose tumours did not grow had a longer survival (14.9 months) (range 1.4–37.2) than those whose tumour could be established (9.1 months) (range 0.2–32.5) (*P*=0.0003).

### Histology after xenograft

**Figure 1:**
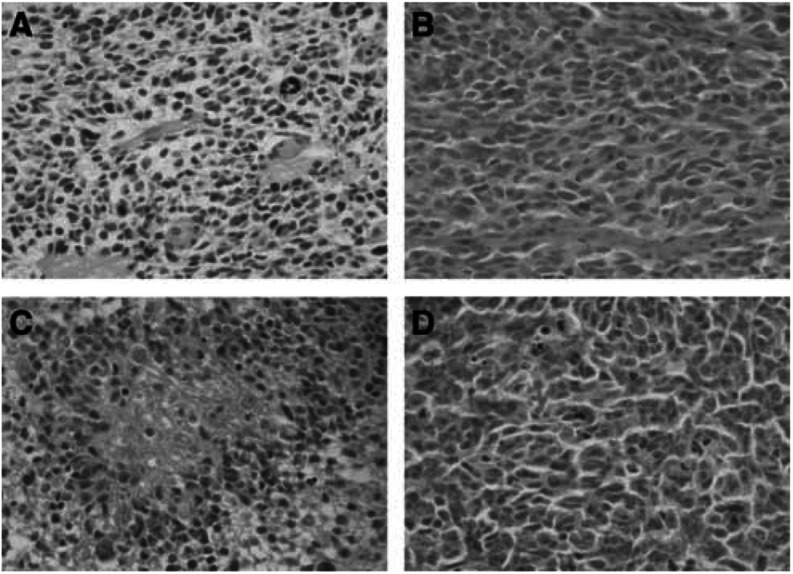
Representative histology of two tumours before and after xenograft on *nude* mice. (**A**) GIR *in situ* tumour, anaplastic oligodendroglioma morphology, haematoxylin–eosin HE staining (magnification × 200); (**B**) ODA-17-GIR corresponding xenograft, passage 1, dedifferentiated morphology, HE staining (× 400); (**C**) ROM *in situ* tumour, GBM histology, haematoxylin–eosin (HE) staining (× 200); (**D**) GBM-7-ROM corresponding xenograft, passage 3, dedifferentiated morphology, HE staining (× 400).

After xenograft in *nude* mice, tumours presented a dedifferentiated phenotype devoid of any particular pattern with small round or fusiform cells. Grafted oligodendrogliomas displayed a complete loss of ‘honeycomb’ appearance (Figure 1B, D

). The cellular density was very high. Neither endothelial hyperplasia nor endothelial proliferation could be observed. Many mitotic and apoptotic figures were seen in the same samples, while central areas of necrosis were almost constant. Tumours lost their glial differentiation whatever their initial type (oligodendroglial or astrocytic), except one tumour with an initial morphology of mixed anaplastic glioma, which exhibited an oligo-like differentiation after xenograft (ABE).

### Genetic alterations of primary tumours and relationship with tumour growth

As summarised in Table 1

**Table 1:** Molecular characterisation of initial tumours

	**Ch1**		**Ch19**	**Ch9**	**Ch10**						
**Tumour**	**P**	**Q**	**Q**	**P**	**P**	**Q**	**TP53**	**PTEN**	**EGFR**	**CDKN2A**	**Telomerase activity**
(a)											
VIL	LOH	LOH	LOH	LOH	LOH	LOH	—	—	amp	hd	ND
RAV	LOH	LOH	—	—	LOH	LOH	mut	—	amp	—	Present
RAI	LOH	LOH	LOH	—	LOH	—	mut	—	—	—	Absent
GEN	LOH	—	LOH	LOH	LOH	LOH	—	—	amp	hd	Present
MEG	LOH	—	LOH	—	LOH	LOH	—	—	—	—	ND
THO	—	—	LOH	LOH	LOH	LOH	mut	mut	—	—	Absent
PID	—	—	LOH	—	LOH	LOH	—	—	—	hd	ND
VIB	—	—	—	LOH	LOH	LOH	—	mut	—	hd	ND
BAS	—	—	—	—	LOH	LOH	—	—	—	—	ND
IOC	—	—	—	LOH	LOH	—	—	—	—	—	ND
MAA	—	—	—		—	—	—	—	amp	—	ND
BOB	—	—	—	—	—	—	—	—	—	—	ND
CAR	—	—	—	—	—	—	mut	—	—	—	ND
KNO	—	—	—	—	LOH	LOH	—	—	—	—	ND
GIR	—	—	—	—	LOH	LOH	—	—	amp	hd	Present
CHA	—	—	—	LOH	LOH	LOH	—	mut	—	hd	Present
HAA	—	—	—	LOH	LOH	LOH	mut	mut	—	hd	Present
ROM	—	—	—	—	LOH	LOH	—	—	—	—	Present
JEU	ND	ND	ND	ND	ND	ND	—	—	amp	hd	Present
											
(b)											
ABD	LOH	—	LOH	LOH	—	LOH	—	—	—	hd	Absent
AMR	LOH	—	LOH	LOH	—	LOH	—	—	—	hd	Present
HAN	LOH	—	LOH	LOH	LOH	LOH	—	—	—	—	Absent
DUR	LOH	LOH	LOH	—	—	—	mut	—	—	—	Absent
MAT	LOH	—	LOH	—	—	—	—	—	—	—	Present
PER	LOH	—	LOH	LOH	—	—	—	—	—	—	Absent
ROU	LOH	—	LOH	—	—	—	—	—	—	—	Present
LAL	—	LOH	—	—	LOH	LOH	—	mut	—	—	Present
GUI	—	—	—	—	LOH	LOH	mut	—	—	—	Present
BEU	—	—	—	LOH	LOH	LOH	—	—	—	—	Absent
JAN	—	—	—	—	—	—	mut	—	—	—	Absent
BOS	—	—	—	—	LOH	—	—	—	—	—	Absent
YBE	—	—	—	—	—	—	—	—	—	hd	Absent
BAY	ND	ND	ND	ND	ND	ND	—	—	—	—	ND
DOR	ND	ND	ND	ND	ND	ND	—	—	—	—	ND
HAD	ND	ND	ND	ND	ND	ND	—	—	—	—	ND
LEB	ND	ND	ND	ND	ND	ND	mut	—	—	—	ND
LOS	ND	ND	ND	ND	ND	ND	—	—	—	—	ND
MAS	ND	ND	ND	ND	ND	ND	—	—	—	—	ND
PET	ND	ND	ND	ND	ND	ND	—	—	—	—	ND
PIG	ND	ND	ND	ND	ND	ND	—	—	—	hd	ND
POU	ND	ND	ND	ND	ND	ND	—	—	—	—	ND
VAU	ND	ND	ND	ND	ND	ND	—	—	—	—	ND
ZAY	ND	ND	ND	ND	ND	ND	—	mut	—	hd	ND

(a) Established tumours; (b) nonestablished tumours; LOH = loss of heterozygosity; mut = mutation; amp = genomic amplification; hd = homozygous deletion; ND = not done.

, screening for p53 mutations, PTEN mutations, EGFR amplifications and p16/INK4a homozygous deletions was performed in 43 tumours. Screening for LOH on chromosomes 1p, 1q, 19q, 10p, 10q, 9p was performed for 31 tumours and for telomerase activity in 22 tumours.

Tumours were separated into two groups. The first one consisted of tumours that grew in *nude* mice for at least two transplantations (*n*=19) and the second group of those that did not (*n*=24). Comparison of the two groups showed that growth in *nude* mice was correlated to 10q LOH and EGFR amplification (*P*=0.01 and 0.0035, respectively). In addition, when genetic alterations linked to anaplasia were pooled (chromosome 10 loss, chromosome 9p loss, EGFR amplification, p16 deletion, PTEN mutation and telomerase reactivation), nongrowing tumours showed significantly fewer alterations than growing tumours (*P*=0.029). Among the whole series, no alteration could be correlated to a nontumorigenic feature.

### Genetic alterations after establishment in *nude* mice

Comparison of genetic alterations was made between primary tumours (at the time of surgery) and first transplantations in *nude* mice, and then between the first and subsequent transplantations. Tumours showed a striking genetic stability before and after establishment in mice (Table 2

**Table 2:** Molecular characterisation before and after establishment on *nude* mouse

	**Ch1**		**Ch19**	**Ch9**	**Ch10**						
**Tumour**	**P**	**Q**	**Q**	**P**	**P**	**Q**	**TP53**	**PTEN**	**EGFR**	**CDKN2A**	**Telomerase activity**
RAI	LOH	LOH	LOH	—	LOH	—	mut	—	—	—	Absent
ODA-5-RAI p1	LOH	LOH	LOH	—	LOH	—	mut	—	—	—	Absent
ODA-5-RAI p4	LOH	LOH	LOH	—	LOH	—	mut	—	—	—	Absent
											
RAV	LOH	LOH	—	—	LOH	LOH	mut	—	amp	—	Present
ODA-14-RAV p1	LOH	LOH	—	—	LOH	LOH	mut	—	amp	—	Present
ODA-14-RAV p13	LOH	LOH	—	—	LOH	LOH	mut	—	amp	—	Present
											
GEN	—	—	LOH	LOH	LOH	LOH			amp	hd	Present
ODA-4-GEN p1	LOH	—	LOH	LOH	LOH	LOH	—	—	amp	hd	Present
ODA-4-GEN p7	LOH	—	LOH	LOH	LOH	LOH	—	—	amp	hd	Present
											
THO	—	—	LOH	LOH	—	LOH	mut	—	—	—	Absent
ODA-20-THO p1	—	—	LOH	LOH	—	LOH	mut	hd	—	—	Absent
ODA-20-THO p6	—	—	LOH	LOH	—	LOH	mut	hd	—	—	Absent

RAI, RAV, GEN, THO = initial tumours. ODA-4-GEN, ODA-14-RAV, ODA-4-GEN, ODA-20-THO = xenografted tumours; p_x_ = passage X; LOH = loss of heterozygosity; mut = mutation; amp = genomic amplification; hd = homozygous deletion; ND = not done.

). No alteration disappeared after xenograft and only two new alterations appeared in xenografts (a 1p loss for ODA-4-GEN and a PTEN homozygous deletion for ODA-20-THO). After the first transplantation, the molecular profile of alterations remained constant.

### Correlations between spontaneous growth rate and genetic alterations

The latency (time between the first transplantation and the appearance of a palpable tumour) and the doubling time for 12 growing tumours are summarised in Table 3

**Table 3:** Growth characteristics of xenografts

	**Latency (in days)**	**Doubling time (in days)**
(a)		
ODA-4-GEN	19	5.5
ODA-5-RAI	33	4.5
ODA-14-RAV	8	5.5
ODA-20-THO	33	6.5
ODA-17-GIR	27	2.5
ODA-25-NAN	101	14
ODA-23-NAN	25	2.5
ODA-24-NAN	66	12
		
(b)		
GBM-21-NAN	46	5.5
GBM-1-HAM	19	5.5
GBM-7-ROM	7	4
GBM-14-CHA	17	3.5

(a) Oligodendroglial xenografts; (b) GBM xenografts. Latency was measured between graft and appearance of a palpable tumour at the first transplantation. Doubling time was measured when it became stable, after passage 5.

showing that even if latency differed between tumours, the doubling time remained relatively stable. For the different genetic alterations tested, multivariate analysis showed that only EGFR amplification was associated with a higher growth rate (*P*=0.0082).

## DISCUSSION

These data indicate that human xenografts are useful models to study the molecular pathways involved in malignant oligodendrogliomas and astrocytomas. However, successful grafting occurred only in anaplastic tumours in terms of pathological grading of the original tumours, genotype analysis and prognostic significance for donor patients.

None of the grafted low-grade gliomas grew on *nude* mice while (45%) 23/51 high-grade gliomas did. Even among malignant gliomas, there was a significant difference, suggesting that higher grade tumours were preferentially selected by the grafting process since 62% (13/21) GBM or GBMO (grade IV) grew as compared to 33% (10/30) AO, AOA (grade III) (*P*=0.0047).

The results of the molecular analysis of the primary tumour (before grafting) are in agreement with the pathological data. Overall, growing tumours had significantly more molecular alterations than the nongrowing ones. This finding is particularly striking for EGFR amplification and LOH on chromosome 10, two alterations highly associated with increased malignancy (Bigner and Vogelstein, 1990; von Deimling *et al*, 1992; Reifenberger *et al*, 1996). It has previously been shown that EGFR amplification gives a tumorigenic advantage in GBM (Humphrey *et al*, 1988; Muleris *et al*, 1994), a feature that we often found associated with stable double-minute extra-chromosomal elements on caryotypic analysis (data not shown) (Bigner *et al*, 1989). In addition, EGFR amplification was present in 4/11 successfully grafted oligodendrogliomas in our series, indicating that the tumorigenic role of this alteration affects various subtypes of gliomas. Interestingly, overexpression of EGFR in transgenic mice has been shown to be involved in tumour progression of oligodendroglioma (Ding *et al*, 2003).

Similarly, we found that LOH on chromosome 10 was also extremely frequent not only in GBM (100%, 7 out of 7) but also in growing oligodendrogliomas (73%, eight out of 11). Thus, the loss of the putative tumour suppressor gene located on chromosome 10 is important for tumorigenicity in both tumour subtypes. However, we did not find evidence of involvement of the PTEN gene, which is located on chromosome10, in this series.

The fact that pathological and molecular factors predictive of successful grafting were associated with higher malignancy explaining that survival was shorter in donor patients whose tumours grew in *nude* mice (9.1 months) as compared to survival of patients whose tumours were rejected (15.4 months) (*P*=0.0003).

**Figure 2:**
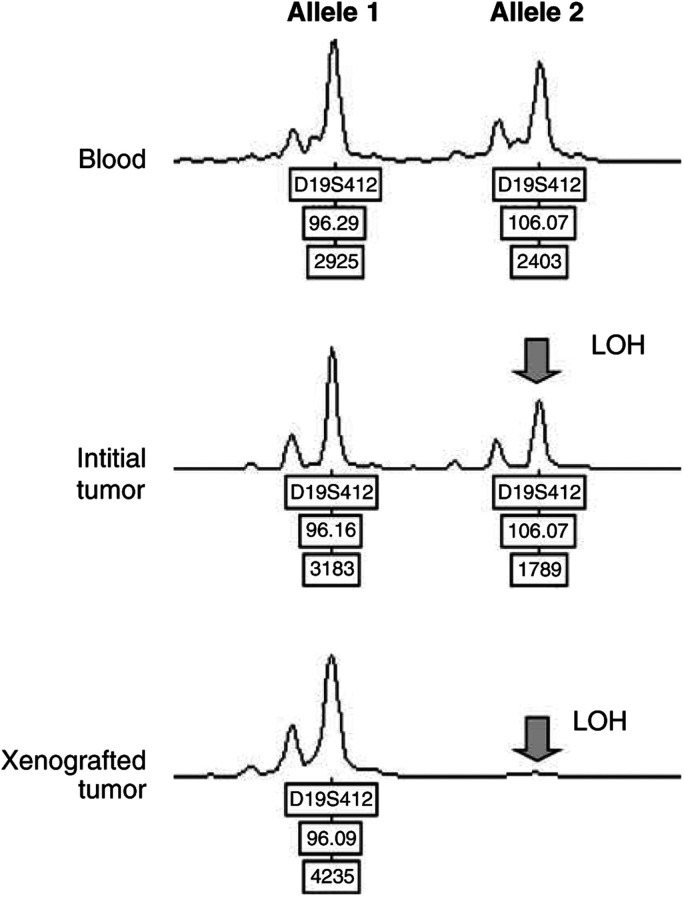
Loss of heterozygosity patterns before and after establishment on *nude* mice. Genescan patterns showed a hardly detectable LOH in the initial tumour, which in contrast appeared very clearly after the first passage onto *nude* mice.

An important characteristic of this model is the stability of the genetic alterations in the xenografts as compared to the primary tumours, a finding also observed after successive passages in nude mice. Such stability, which was somewhat controversial in previous studies (Bigner *et al*, 1989; Muleris *et al*, 1994; Goike *et al*, 1999; Jeuken *et al*, 2000), appears to hold both for GBM and anaplastic oligodendrogliomas. Nevertheless, rare differences before and after growth on nude mice were shown as illustrated in Table 2. Rather than selection of a minor subclone, it is likely, as shown in Figure 2

, that these alterations were already present in the original tumour, but were hardly visible or even undetectable because of the presence of ‘contaminating’ normal tissue.

An advantage of this model is therefore to obtain tumours devoid of normal cells, improving the quality of molecular analyses (Figure 2).

Despite a stable genetic profile, the phenotype of the grafted tumour cells underwent striking changes whatever the histological features of the original tumour. After xenograft, all but one glioma lost their glial differentiation and presented a dedifferentiated phenotype in contrast with xenografts derived from other human cancers that maintained several of their histological characteristics (Rofstad *et al*, 1990a, 1990b; Poupon *et al*, 1993; Kolfschoten *et al*, 2000; Bras-Goncalves *et al*, 2001; de Pinieux *et al*, 2001; Krasagakis *et al*, 2001).

Whether the morphological homogeneity of grafted tumours represents a nonspecific change or supports the view that many gliomas have a common cell of origin (Daumas-Duport *et al*, 1997) leading to various morphological appearance in patients remains unsettled.

In summary, xenografting malignant astrocytomas and oligodendrogliomas is a useful method for studying and probably refining knowledge of the spectrum of genetic alterations in these tumours. This method can be of help in analysing the correlations between genotype and response to chemotherapy or various experimental agents. In addition, it may further benefit from new approaches such as gene expression by microarray studies in order to identify genes associated with chemosensitivity.
